# A Cross-Sectional Survey to Evaluate Potential for Partnering With School Nurses to Promote Human Papillomavirus Vaccination

**DOI:** 10.5888/pcd17.190451

**Published:** 2020-09-24

**Authors:** Matthew Bozigar, Trevor D. Faith, Ashley A. White, Ka’la D. Drayton, Allison Fabick, Kathleen B. Cartmell

**Affiliations:** 1 Department of Public Health Sciences, Medical University of South Carolina, Charleston, South Carolina; 2 Biomedical Informatics Center, Medical University of South Carolina, Charleston, South Carolina; 3 College of Medicine, Medical University of South Carolina, Charleston, South Carolina; 4 College of Pharmacy, Medical University of South Carolina, Charleston, South Carolina; 5 Department of Public Health Sciences, Clemson University, Clemson, South Carolina

## Abstract

**Introduction:**

The human papillomavirus (HPV) increases the risk for cancers of the cervix, oropharynx, vulva, vagina, penis, and anus. HPV vaccination rates are low in many states having large medically underserved areas. In such areas, school nurses are a potential partner for improving population health, but their perceptions about HPV, HPV vaccination, and their role in promoting HPV vaccination have not been well documented.

**Methods:**

We administered a cross-sectional survey to 61 of 74 lead school nurses at their 2019 annual training session in South Carolina. Survey questions assessed lead school nurses’ HPV vaccination beliefs, barriers, and HPV vaccination role in schools. We tabulated descriptive data and created heat maps to visualize correlations between responses.

**Results:**

Despite 95.1% of nurses envisioning a role in supporting HPV vaccination at their schools, only 41.0% envisioned an active role in promoting HPV vaccine among students. Lead nurses consistently believed in vaccinating both male and female students; in vaccine safety, effectiveness, and health benefits; and in recommending HPV vaccination. The nurses agreed that lack of time and competing priorities were barriers to HPV vaccination. Few other barriers were consistently identified.

**Conclusion:**

Partnering with school nurses may be a feasible strategy to overcome barriers to increasing HPV vaccination rates in medically underserved areas. However, to increase nurses’ confidence and time allotment to assume an active role in HPV vaccine promotion in their schools, coordinated and sustained partnerships between public health agencies, school districts, and school nurses are needed.

**Summary**

**What is already known on this topic?**

School nurses, as trusted community health professionals, are essential partners for improving adolescent human papillomavirus (HPV) vaccination coverage. Barriers to HPV vaccine promotion, school nurses’ knowledge, and their perceived roles have been studied in some populations.

**What is added by this report?**

Among lead school nurses in South Carolina, a state with large medically underserved areas, the lack of confidence and time to interact with students and parents are key hindrances to HPV vaccine promotion.

**What are the implications for public health practice?**

Although school nurses have many competing work priorities, opportunities exist to engage them in supporting coordinated public health programs aimed at improving HPV vaccination rates in medically underserved areas.

## Introduction

Human papillomavirus (HPV) is an oncogenic, sexually transmitted infection (1). HPV is responsible for an estimated 42,700 cancer cases each year in the United States, comprising 90% of anal/cervical cancers, 70% of vaginal and vulvar cancers, and 60% of penile cancers (2). The HPV vaccine is effective, safe, and recommended for adolescent boys and girls aged 11 and 12, whether they are sexually active or not (3,4).

Despite recommendations, vaccine uptake varies widely across geographic areas. An estimated 42.7% of adolescents aged 13 to 17 in South Carolina have completed the HPV vaccination series, below the national average of 51.1% (5). Similarly, most southern states have HPV vaccination rates below the national average (6), but school-based, physician-led interventions have successfully improved vaccination coverage in medically underserved areas (7). Of the 46 counties in South Carolina, 29 entire counties and many parts of the remaining counties are medically underserved (8). As such, additional evidence-based research is needed to inform strategies to improve HPV vaccination rates in medically underserved areas (9).

Given their role and everyday contact with students and parents, school nurses are some of the most trusted health professionals in local communities (10). Consequently, school nurses are invaluable local health resources and potential health advocates. Previous research identified wide support for in-school vaccination (11), in addition to outreach by school nurses to parents and students as a practical leadership role in supporting HPV vaccination (12). But the extra workload can also be a barrier (13).

Lead school nurses are an important group to study, because they oversee the nurses working in their school districts. Each is responsible for the nursing leadership of several schools. Duties of lead school nurses include ensuring district-wide compliance with state policies, acting as advocates for stakeholders and their constituent nurses, and maintaining staffing for schools in their districts. Such nurses often oversee schools at multiple levels of education (ie, elementary, middle, and/or high school).

Our objectives were to identify lead school nurses’ perceptions about HPV vaccination and about nurses’ role in supporting HPV vaccination in a largely medically underserved population. To address these objectives, we conducted a cross-sectional survey with lead school nurses in South Carolina to identify potential strategies to facilitate HPV vaccination and increase population vaccine coverage.

## Methods

Lead school nurses were surveyed at their annual training meeting on February 14, 2019, in Columbia, South Carolina, by using a cross-sectional design. The inclusion criterion was currently being a lead school nurse in South Carolina; we had no exclusion criteria. Our sampling frame was all lead school nurses present at the annual meeting, and the target population was school nurses currently employed in South Carolina.

We adapted a previously published survey (14) to assess lead school nurses’ beliefs, perceived roles, and barriers to advocacy and education in school settings. Although to our knowledge the instrument has not been validated, few, if any, validated questionnaires are available to assess school nurse perceptions about HPV vaccination. The initial version of the present survey was pilot-tested at the 2019 Annual School Nurses Conference in Columbia, South Carolina, attended by more than 200 school nurses from throughout the state. Feedback on survey design and composition was elicited from 30 of the nurses via convenience sampling. We used their feedback to inform the design of the final survey. Feedback indicated the need for several changes, such as including additional professional practice options, including additional response types for questions about barriers, shortening the survey, and modifying the structure of some questions for usability and clarity. The institutional review board at the Medical University of South Carolina (MUSC) declared this study exempt from review, because the primary purpose of the survey was to inform public health activities (for quality improvement) and the survey collected no personal identifiers.

Beliefs about HPV and HPV vaccinations were assessed by using a 5-point Likert scale. Twelve questions were asked in this domain, which assessed the nurses’ knowledge about the safety and effectiveness of the vaccine, in addition to their perceived confidence in disseminating that information. Responses to questions in this domain ranged from 1 (strongly disagree) to 5 (strongly agree), with 3 as a neutral option. Nurse’s professional practices were documented by using a checklist of activities they performed in their schools, including advocating for and educating about HPV vaccination, and partnering with health agencies. A 5-point Likert scale, with response options ranging from 1 (never) to 5 (always) was used to assess how often the nurses experienced barriers to advocacy and education. Eleven distinct barriers were assessed, including the nurse’s own knowledge, opportunities to talk to students/parents, policies, and personal beliefs about the vaccine. Finally, 3 questions were designed to determine the nurses’ confidence and ability to disseminate vaccine information. A 3-point Likert scale was used for this domain, which included response options of 1 (not confident), 2 (somewhat confident), and 3 (very confident).

Lead nurses completed the survey by using paper copies and pen or pencil, during a break in the conference program. The survey was completed by 61 of 74 (82.4%) lead school nurses in attendance, although not all answered every survey question. The lead nurses represented South Carolina’s 79 public school districts, as well as lead nurses or nurses working alone in private, parochial, charter, and independent schools. Approximately 150 lead school nurses (all those known by the South Carolina Department of Health and Environmental Control) were invited to the conference. Therefore, 40.7% of the population of all South Carolina lead school nurses completed the survey.

Data were organized and analyzed by using SPSS Statistics version 25 (IBM Corporation) and R version 3.5.0 (R Foundation for Statistical Computing) software packages. Descriptive data were analyzed by using SPSS. Response frequencies and corresponding proportions (percentages) were calculated for each question. Response categories for questions 3 (perception) and 5 (barriers) were each collapsed (eg, combined strongly agree with agree, always with often), to better represent the distribution of responses. R was used to create heat maps of the correlations between response values. We used Spearman correlations that used ranked values, which are suitable for noncontinuous variables, instead of Pearson correlations that assume linear relationships for continuous variables. Correlation values can range from −1 (perfectly negatively correlated) to 1 (perfectly positively correlated). In these heat maps, deeper shades of blue indicate responses that were strongly negatively correlated (ie, more dissimilar) and deeper shades of red indicate responses that were strongly positively correlated (ie, more similar). A clustering algorithm used in the *hclust* function in R provided the structure for grouping similar correlations together in the heat map. Fundamentally, a heat map is a correlation table, but it enhances the ability to identify particularly strong and weak correlations. However, correlations should not be interpreted as causative.

## Results

### Beliefs

Nearly all respondents agreed that male students (93.4%), female students (93.3%), and preteens (91.5%) should be vaccinated against HPV (Table 1). Similarly, nearly all respondents agreed that for most people the vaccine is safe (90.2%), nontoxic (88.3%), and that it prevents HPV cancers (93.3%). However, for most of the remaining belief statements, responses were more varied. This set of more controversial statements (between 33% and 67% agreement) included “I can influence parents,” “Parents will vaccinate if given information,” “I am a leader in providing vaccine information,” “I can permissibly provide information to parents,” “I can permissibly provide information to students,” and “Information I disseminate must have district approval.”

**Table 1:** Lead School Nurses’ Beliefs About Human Papillomavirus (HPV) and Vaccination, Cross-Sectional Survey of Lead School Nurses, South Carolina, 2019

Belief	Strongly Disagree/Disagree, No. (%)	Neutral, No. (%)	Strongly Agree/Agree, No. (%)
I believe HPV is harmful to a person’s health	12 (19.7)	2 (3.3)	47 (77.0)
I believe male students should be vaccinated against HPV	0	4 (6.6)	57 (93.4)
I believe female students should be vaccinated against HPV	0	4 (6.7)	56 (93.3)
I believe preteens should receive the HPV vaccine before they become sexually active	0	5 (8.5)	54 (91.5)
I believe the HPV vaccine is safe for the majority of the population	0	6 (9.8)	55 (90.2)
I believe the HPV vaccine is nontoxic for the majority of the population	0	7 (11.7)	53 (88.3)
I believe the HPV vaccine prevents HPV-related cancers	0	4 (6.7)	56 (93.3)
I believe by providing my professional opinion, I can influence parents to vaccinate their child/adolescent against HPV	1 (1.7)	18 (30.0)	41 (68.3)
I believe if I provide information to parents, they will vaccinate their child/adolescent against HPV	4 (6.6)	34 (55.7)	23 (37.7)
I can see myself as a leader in providing HPV vaccine information in the school community	4 (6.6)	23 (37.7)	34 (55.7)
I can provide health education to parents without violating school district policy	5 (8.2)	15 (24.6)	41 (67.2)
I can provide health education to STUDENTS without violating school district policy	8 (13.3)	20 (33.3)	32 (53.3)
Any health information I disseminate to STUDENTS or PARENTS must have approval from the school district	8 (13.3)	6 (10.0)	46 (76.7)

**Figure 1:**
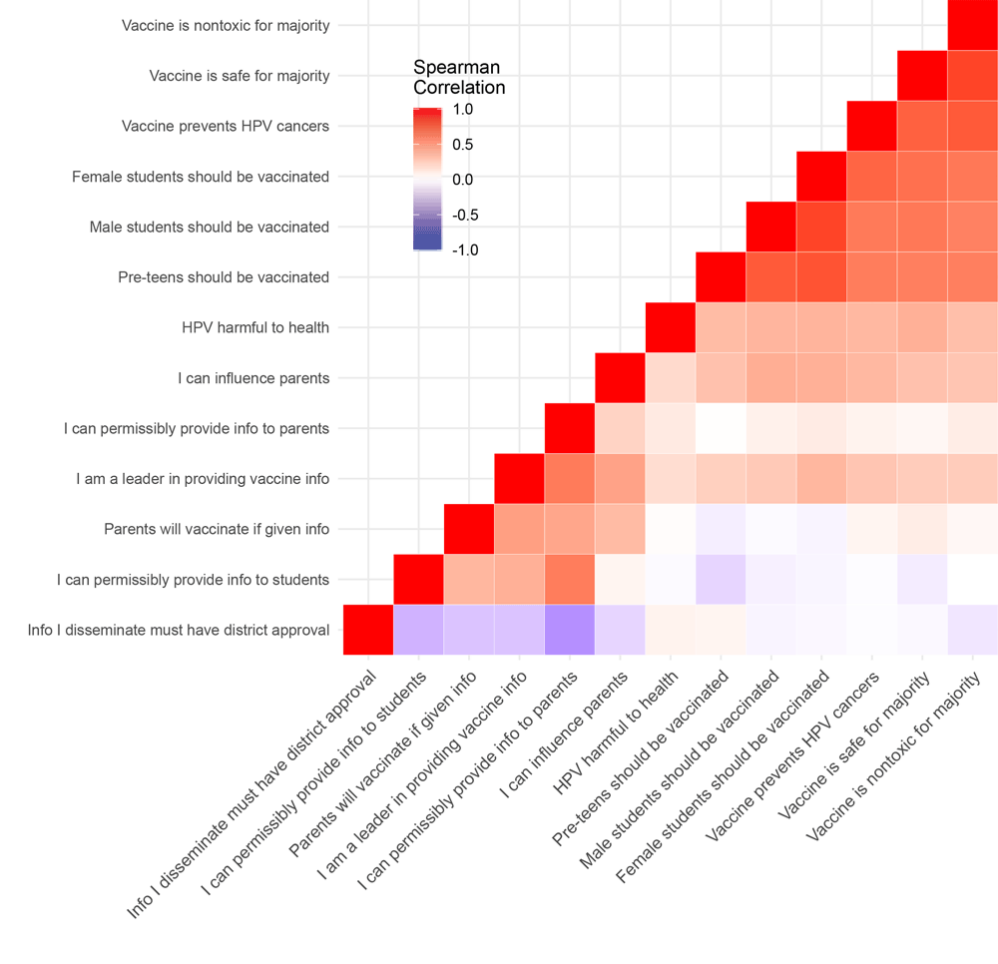
Heat map indicating Spearman correlation values for responses to survey question about human papillomavirus (HPV) and vaccination beliefs.

The responses to HPV vaccination guidelines, safety, and effectiveness were highly correlated (Figure 1). For example, a lead school nurse who agreed with the statement that preteens should be vaccinated also was highly likely to agree with the statement that the HPV vaccine is nontoxic, just as they would agree with the remainder of the statements about benefits, guidelines, safety, and effectiveness.

Other patterns indicated that responses were less correlated or even inversely correlated (Figure 1). A unique set of relationships occurred with the “Information disseminated requires district approval” statement, which was not strongly correlated with the statements on benefits, guidelines, safety, and effectiveness (indicated by neither red nor blue shading); and it was inversely correlated (ie, answered dissimilarly, indicated by blue shading) with the statements about HPV vaccination leadership, permissibility, parent influence and action, and information dissemination. As such, a lead school nurse that agreed with the statement that “Information disseminated requires district approval” was no more or less likely to agree with beliefs about benefits, guidelines, safety, or effectiveness, and less likely to agree with statements that they are HPV leaders, can permissibly provide information, or can influence parent action.

### Role

Ninety-five percent of lead school nurses thought they should play a role in HPV vaccination (Table 2). However, only 41.0% of the nurses endorsed having a proactive role in talking with students about HPV vaccination and only 27.9% endorsed conducting HPV educational events. Conversely, most nurses supported having a role in answering questions about HPV vaccinations from students (68.9%), and even more from parents (82.0%). School nurses endorsed taking an active role in talking with parents about the HPV vaccine (60.7%) to a greater extent than having this same conversation with students. Most nurses were only somewhat confident in their knowledge about HPV when talking either to parents (55.7%) or students (57.4%).

**Table 2:** Lead School Nurses’ Perceived Role in Human Papillomavirus (HPV) Vaccination Education and Implementation, Cross-Sectional Survey of Lead School Nurses, South Carolina, 2019

Role	No. (%) (N = 61)
Answering questions about HPV vaccination when students ask	42 (68.9)
Actively talking with students about HPV vaccination	25 (41.0)
Answering questions about HPV vaccination when parents ask	50 (82.0)
Actively talking with parents about the HPV vaccination	37 (60.7)
Conducting educational events to promote HPV vaccination at your schools	17 (27.9)
Partnering with DHEC to provide vaccination clinics in schools	30 (49.2)
Educating parents by answering their questions and providing information	46 (75.4)
School nurses should not play a role in HPV vaccination	3 (4.9)

Abbreviation: DHEC, Department of Health and Environmental Control.

### Barriers

**Figure 2:**
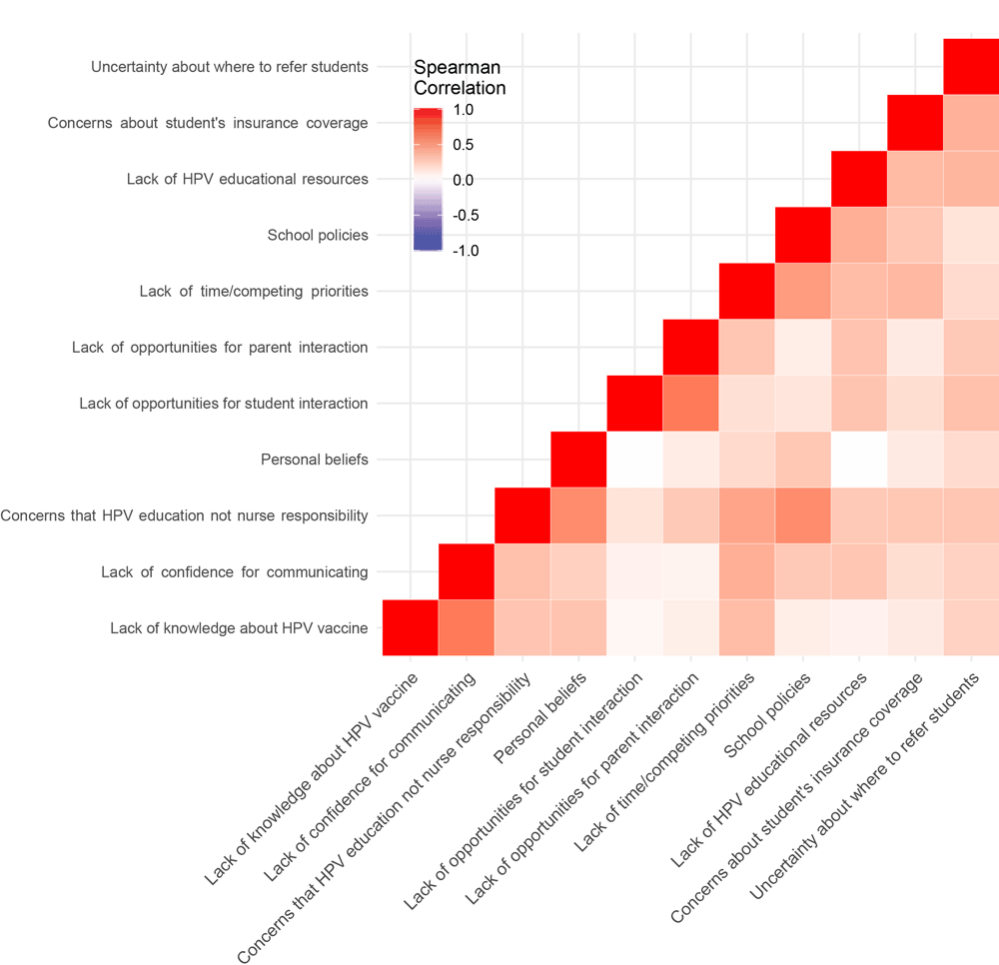
Heat map indicating Spearman correlation values for responses to survey question about barriers to vaccination promotion for human papillomavirus (HPV).

Lead school nurses responded to statements about barriers to HPV vaccine promotion (Table 3, Figure 2). A constant barrier was lack of time or competing priorities (75.0%). The next most common barriers identified, though much less consistently, were concerns about students’ insurance coverage (39.7%) and school policies (38.3%). Some of the barriers encountered least frequently were uncertainty about where to refer students (13.3%), lack of opportunities for student interaction (16.7%), concerns that HPV education is not the nurse’s responsibility (22.4%), and personal beliefs (13.8%).

**Table 3:** Perceived Barriers to Lead School Nurses Providing Human Papillomavirus (HPV) Vaccine Education and Recommendation, Cross-Sectional Survey of Lead School Nurses, South Carolina, 2019

Barrier	Never/Rarely, No. (%)	Sometimes, No. (%)	Often/Always, No. (%)
Lack of knowledge about HPV vaccination	18 (30.0)	27 (45.0)	15 (25.0)
Lack of confidence in how to talk with students and parents about HPV vaccination	15 (25.0)	25 (41.7)	20 (33.3)
Lack of time/competing priorities	2 (3.3)	13 (21.7)	45 (75.0)
Concern that students may not have insurance coverage for HPV vaccination	9 (15.5)	26 (44.8)	23 (39.7)
Uncertainty where to refer students for HPV vaccination if they do not have a regular provider	35 (58.3)	17 (28.3)	8 (13.3)
Lack of HPV vaccination educational resources	18 (30.5)	21 (35.6)	20 (33.9)
Lack of opportunity to interact with students	29 (48.3)	21 (35.0)	10 (16.7)
Lack of opportunity to interact with parents	15 (25.0)	26 (43.3)	19 (31.7)
Concern that HPV vaccination education is not the school nurse’s responsibility	25 (43.1)	20 (34.5)	13 (22.4)
School policies	13 (21.7)	24 (40.0)	23 (38.3)
Personal beliefs	30 (51.7)	20 (34.5)	8 (13.8)

## Discussion

As trusted sources for health information, school nurses play an integral role in community health. However, little is known about the role of school nurses in facilitating HPV vaccinations in states with many medically underserved areas. Our research sought to identify beliefs, roles, and barriers to HPV vaccination promotion among lead school nurses in South Carolina. We highlighted common perceptions about HPV, HPV vaccination, and the potential role in HPV vaccine promotion among lead school nurses who represented districts comprising elementary, middle, and high schools. Demographic data were not collected, and data on the broader school nurse population in South Carolina were unavailable. However, researchers have shown that 246 of 424 zip codes and approximately 43% of the population aged 20 years or older were medically underserved in South Carolina in 2010 (15). As such, school nurses support the health of students from many medically underserved areas in South Carolina.

Our findings were only somewhat consistent with the handful of studies that examined factors among school nurses that affect HPV vaccine uptake. Our study showed correlation between HPV knowledge, HPV vaccine knowledge, and attitudes toward HPV. Consistent with our results, another study reported that school nurses’ positive attitudes about the HPV vaccine were associated with both knowledge about HPV and the HPV vaccine (12). Our results showed that most lead school nurses believed the HPV vaccine should be given to male and female preteens and that the HPV vaccine was safe, nontoxic, and prevents HPV cancers. Yet these underlying beliefs were not correlated with either endorsement of being a leader in providing HPV vaccine information or belief that parents would allow their children to be vaccinated based on nurses’ recommendations. Similar to our study, another reported that knowledge and attitudes were not associated with school nurses’ providing HPV information to parents in Missouri (16). Although several studies reported an association between positive HPV vaccination attitudes and having roles as HPV vaccine opinion leaders (12,17), we did not observe this association in our study. Our review of these studies shows some evidence that HPV vaccine education can improve attitudes about the vaccine and confidence in speaking with students and parents about HPV vaccination. Schools could consider options, such as integrating school nurse–led HPV vaccine education into large group meetings with parents and guardians (eg, during orientation, parent–teacher association meetings, or graduation ceremonies).

Researchers in another study surveyed 145 school nurses in Ohio and found that HPV knowledge had only small positive effects on intention and self-efficacy to become a vaccine champion, and that attitudes had larger positive effects on both intention and actual professional HPV practices (eg, communicating HPV information to parents) (14). The study identified a key pathway stemming from self-efficacy that had moderate effects on both intention and professional practice (14). In our population, correlated responses from school nurses about barriers such as lack of time (because of competing priorities), lack of confidence for communicating, lack of knowledge about the HPV vaccine, and concerns that HPV education is not a school nurse’s responsibility together seem to indicate low self-efficacy of South Carolina school nurses for being HPV vaccination promoters. Advocating to school boards for adequate staffing levels of school nurses could increase the time for HPV vaccine education by decreasing competing priorities, which could, in turn, improve HPV vaccination promotion self-efficacy.

The strong positive correlation we found between the perceived barriers of school policies and “concerns that HPV education is not the nurse’s responsibility” suggests that more research is needed into administrative barriers if school nurses are to have roles as HPV vaccine advocates. Although lead school nurses did not strongly identify with the perceived role of leading vaccination clinics in schools, one study found that both parents and care providers supported providing HPV vaccinations in schools, citing reasons of convenience, improved access, reduced administrative burden, and positive peer pressure (11). However, that study lacked information about its sampling scheme and was limited to one undefined inner-city clinic; therefore, it is not widely generalizable. Yet a promising study in a rural medically underserved community in Texas using a quasi-experimental design found that a physician-led HPV education and immunization program boosted HPV vaccination coverage (7). However, some school nurses in the United Kingdom cited poor relationships with their schools and uncooperative or indifferent school attitudes as barriers to administering the Cervarix HPV vaccine (GlaxoSmithKline) in their schools (13). In the United States, the degree to which school districts and individual schools adopt and support the recommendations of the Advisory Committee on Immunization Practices (ACIP) may vary (18). And although another study in the United Kingdom found that the in-school HPV vaccination program exceeded expectations, it came at the expense of time formerly dedicated to providing one-on-one support to vulnerable students (19). Implementing such a resource-intense and coordinated strategy in South Carolina’s medically underserved areas may be desirable for some of the benefits previously demonstrated elsewhere, but it may be currently infeasible on a population scale without a highly coordinated intrastate school health campaign and additional resources (13). As such, given our results in South Carolina, initial interventions in that state may need to prioritize increasing school nurse confidence and time allotted to HPV vaccination promotion over introducing in-school vaccination interventions, which could be a future priority. To overcome barriers, sustained partnerships aimed at increasing administrative support might be needed. However, given that school nurses are largely already overburdened, administrative (ie, “top-down”) prioritization may be needed to clarify time allotment, administrative directives such as adopting ACIP recommendations, and continued HPV education to empower and engage school nurses as key partners in local communities.

Our study had limitations. The data were self-reported, thus the potential exists for socially desirable responses and recall biases. However, the surveys were completed anonymously, which may have helped to reduce the potential to provide desirable responses. The data were cross-sectional, so any causal inference and generalization to national school nurse populations should be made with great caution. We emphasize that correlation is not causation, but correlations can be indicative of associations warranting further investigation to understand potential causality. The lack of demographic information for respondents also hinders generalizability. Thus, the sample assessed in our study may not be representative of all lead school nurses in South Carolina. Despite these limitations, the strengths of our study include broad representation of lead school nurses from elementary, middle, and high schools across South Carolina. These findings provide insight into perceived roles of lead school nurses and barriers to HPV vaccination in schools in South Carolina, a state with many medically underserved areas, similar to other southern states. Therefore, these findings could be useful for future research, informing policy, and guiding resource allocation to improve vaccination rates across the state and in similar states.

By using a cross-sectional survey, we identified perceptions, roles, and barriers to HPV vaccination promotion among lead school nurses in South Carolina. We analyzed results and correlations between survey responses by using heat maps, which allowed us to compare the consistency of school nurses’ responses. The nurses identified key HPV vaccination promotion barriers including lack of time, competing priorities, and lack of self-confidence. We further identified multiple potential strategies to overcome the persistently identified barriers, including improving HPV knowledge, attitudes, and self-efficacy, and addressing administrative policies and support for school nurses. Improved support for school nurses could empower and engage them as partners in reducing HPV vaccination disparities in their communities. Although these results are unique to South Carolina, many other similar states, particularly in the southern United States, may benefit from the knowledge gained from this study. However, continued research is needed into local barriers to HPV vaccination and opportunities to overcome them nationwide.
